# Atrioventricular Nodal Reentrant Tachycardia Ablation Using Mini-Electrode Recordings

**DOI:** 10.3390/jcm11010282

**Published:** 2022-01-05

**Authors:** Nicolas Clementy, Gérôme Pineaud, Arnaud Bisson, Dominique Babuty

**Affiliations:** Electrophysiology Laboratory, Department of Cardiology, Trousseau Hospital, University of Tours, 37044 Tours, France; ggeromep@hotmail.fr (G.P.); arnaud.bisson37@gmail.com (A.B.); d.babuty@chu-tours.fr (D.B.)

**Keywords:** atrioventricular nodal reentrant tachycardia, radiofrequency ablation, slow pathway, double potential, conduction block, mini-electrode

## Abstract

Catheter ablation of the slow pathway in patients with atrioventricular nodal reentrant tachycardia (AVNRT) is mainly performed using anatomical landmarks. We sought to see whether a new ablation catheter equipped with mini-electrodes may facilitate the mapping of slow pathway potentials for AVNRT ablation. We prospectively included patients referred for AVNRT in our center. Mapping and ablation were performed using an irrigated catheter equipped with 3 insulated mini-electrodes on the distal tip. Thirteen consecutive patients were included (85% female, median age 46 years). Slow pathway potentials could be identified in 77% of cases on mini-electrode bipolar tracings, versus 15% on conventional bipolar tracings (*p* = 0.0009). At the end of the procedure, double potentials on the ablation line were identified in all patients, only on mini-electrode bipolar tracings. Following ablation, an interval separating double potentials in sinus rhythm ≥15% of baseline tachycardia cycle length was associated with non-inducibility in all patients (*p* < 0.0001). No recurrence occurred during 1 year of follow-up. The use of mini-electrodes may help target slow pathway potentials during AVNRT ablation. Identification of sufficiently split double potentials on the ablation line might represent an electrophysiological endpoint in these patients.

## 1. Introduction

Atrioventricular (AV) nodal reentrant tachycardia (AVNRT) is the most frequent junctional tachycardia. Catheter ablation of the slow pathway can be recommended in symptomatic patients as it is an efficient and permanent therapy with a >90% success rate [1]. During the procedure, the mapping of the area of interest is mainly driven by anatomical landmarks (at the basal portion of the Koch triangle, anterior to coronary sinus ostium) rather than electrophysiological criteria. More specifically, discrete sharp and discrete potentials initially described by Jackman and Haissaguerre, respectively, are not routinely used to target the slow pathway [2,3]. These signals are indeed challenging to identify, and their pathophysiological significance remains unclear.

We sought to see whether the use of a new ablation catheter with a distal tip equipped with mini-electrodes could help map the AV nodal slow pathway and thus provide an electrophysiological endpoint for ablation of AVNRT.

## 2. Materials and Methods

### 2.1. Population

We prospectively included consecutive patients addressed for an electrophysiological study in our department with a final diagnosis of AVNRT. Patients <18 years were excluded.

The local ethics committee for human research approved the study protocol. All patients signed informed consent before inclusion.

Demographic characteristics as well as data on the presence of a structural heart disease, the clinical presentation and the antiarrhythmic management were collected.

### 2.2. Ablation Procedure

Surface and endocardial tracings recordings were performed on a dedicated electrophysiological recording platform (LabSystem Pro 9900 with dedicated software version 2.7a, Bard Electrophysiology, Boston Scientific, Lowell, MA, USA). The filter settings were 30–250 Hz (notch filter 50 Hz). Through a right femoral venous approach, a decapolar deflectable catheter was positioned within the coronary sinus (CS) with the proximal bipole 9–10 at the ostium, a quadripolar catheter at the His bundle, and a quadripolar catheter moved either at the high right atrium or at the right ventricular apex.

Atrial and ventricular incremental pacing, as well as extrastimuli, were used to identify electrophysiological properties of the AV nodal slow pathway, and to induce sustained AVNRT. Atropine injection (1 milligram bolus) and/or isoproterenol infusion were eventually performed to promote tachycardia induction when necessary.

**Figure 1:**
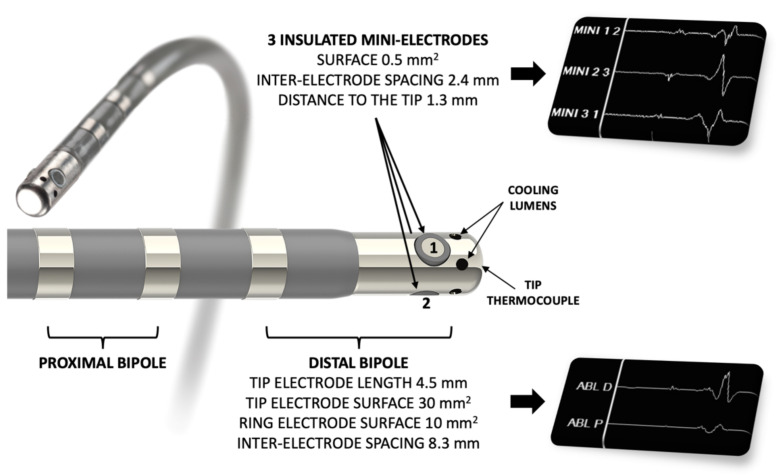
Catheter used for mapping and radiofrequency ablation (IntellaTip Micro Fidelity Open Irrigated MiFi OI, Boston Scientific, Boston, MA, USA). The three insulated mini-electrodes are radially arranged directly on the radiofrequency-delivering 4.5 mm irrigated distal tip. Bipolar recordings are performed between electrodes 1–2, 2–3 and 3–1.

A 7.5 F irrigated-tip radiofrequency (RF) ablation catheter was used to map the AV slow pathway, equipped with three insulated 0.5 mm^2^ “mini-electrodes” (IntellaTip Micro Fidelity Open Irrigated MiFi OI, model M004EPM9620N40, Boston Scientific, Boston, MA, USA) (Figure 1).

Mapping was performed anteriorly to the coronary sinus ostium, and radiofrequency energy was applied during 60 s (power setting 30 W, temperature limited to 45 °C) where slow potentials could be identified. Early far-field signal followed by a late relatively high-amplitude near-field signal was identified as a “Jackman” J potential, and an early high-amplitude near-field signal followed by a far-field component as a “Haissaguerre” H potential [2,3]. Inducibility was tested after each radiofrequency energy application.

All tracings were reviewed by two experienced electrophysiologists (NC and DB).

### 2.3. Follow-Up

All patients underwent a telephone interview at 1 year to assess clinical status.

### 2.4. Statistical Analyses

Analyses were performed using JMP software (version 9.0, SAS Institute, Cary, NC, USA). Quantitative variables were expressed as median, range and interquartile range (IQR). Comparisons between groups were performed using non-parametric tests. A *p*-value < 0.05 was considered significant.

## 3. Results

### 3.1. Population

A total of 13 consecutive patients addressed for ablation of symptomatic AVNRT were prospectively included. Baseline characteristics are reported in Table 1. They were index procedures for all patients but one (#13).

### 3.2. EP Study

Detailed electrophysiological characteristics are reported in Table 2. The total procedure median duration (including ablation) was 66 min (range 51–131, IQR 41). Baseline median AH, HV interval, and anterograde atrioventricular block were 108 (range 72–164, IQR 25), 50 (range 32–72, IQR 13), and 333 (range 261–600, IQR 71) milliseconds (ms), respectively. Induction of typical AVNRT (slow-fast type in all patients) was performed without the need of any medication in eight patients, under isoproterenol alone in three, and under both isoproterenol and atropine in two. The median tachycardia cycle length (TCL) was 350 ms (range 230–415, IQR 87).

**Figure 2:**
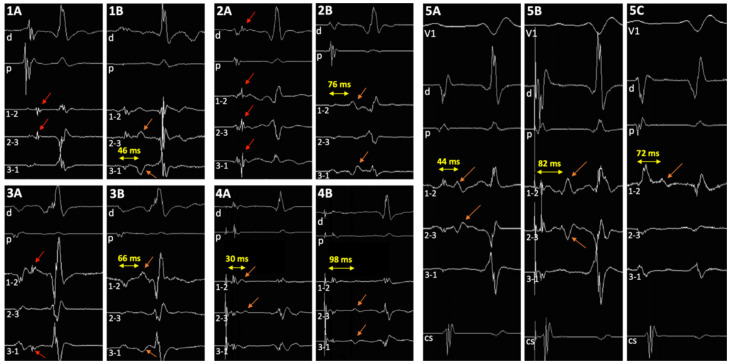
Tracings in 5 patients. (**1A**–**3A**) Sharp potential as described by Jackman and colleagues can be easily identified before ablation on the mini-electrodes bipolar channels, and on distal ablation bipole in 1 patient (**2A**) (red arrows); (**1B**–**3B**) post-ablation double potentials can be visualized (orange arrows). (**4**) Discrete low dV/dt potential as described by Haissaguerre and colleagues before ablation (**4A**), and post-ablation double potentials (**4B**) (orange arrows). (**5**) Haissaguerre slow pathway potential before ablation in sinus rhythm (**5A**) and during atrial pacing (**5B**), and post-ablation double potentials (**5C**) (orange arrows). V1: precordial surface lead V1; d and p, ablation catheter distal (1–2) and proximal (3–4) bipolar recordings, respectively; 1–2, 2–3, and 3–4, bipolar channels between corresponding mini-electrodes.

A sharp potential, as initially described by Jackman and colleagues, was identified in three patients on mini-electrodes bipolar signals, in one patient on conventional electrodes (Figure 2) [3]. A low dV/dt potential, as initially described by Haïssaguerre and colleagues, was identified in eight patients on mini-electrodes signals, and only in two patients on conventional bipolar signals [2]. Overall, specific potentials, identified as potential slow pathway potentials, were identified in 77% of cases on mini-electrode bipolar tracings versus 15% on conventional bipolar tracings (*p* = 0.0009).

### 3.3. Ablation

A median of 4 (range 2–21, IQR 11) RF applications of 60 s were performed. The irrigation rate was set to 15 mL/min, with a temperature limit of 45 °C. Power was set at 20 W during 10 s, then increased up to 30 W during the remaining application time. An escape junctional rhythm during application was observed in 69% of cases. No patient was inducible at the end of the procedure. Only one patient had one persistent isolated echo beat.

### 3.4. Follow-Up

All patients were discharged the same day, without complication. No recurrence was observed at 12 months.

### 3.5. Double Potentials

**Figure 3:**
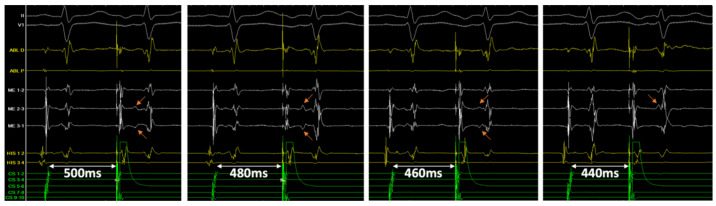
Tracings in patient #11 following successful ablation of the slow pathway. Double potentials can be easily identified on the mini-electrodes bipolar channels (ME 2–3 and 3–1), not on the distal ablation conventional bipolar tracing (ABL D). From left to right, with an atrial extra-stimulus coupling interval of 500, 480, 460 and 440 ms, the delay between sharp and slow potential (orange arrows) progressively increases from 114 to 180 ms, until it is hidden into the ventricular farfield potential.

Identification of a double potential before ablation was possible in 69% of cases, only on mini-electrode bipolar signals: in 6 cases in sinus rhythm, with a median interval separating potentials of 39 ms (range 28–52, IQR 17), and in three additional cases only during pacing of the high right atrium at 600 ms, with a median delay of 34 ms (range 25–61, IQR 29). After ablation, a double potential was identified in 100% of patients, only on mini-electrodes bipolar tracings (*p* = 0.0002): in sinus rhythm in 10 patients, with an interval of 62 ms (range 30–76, IQR 17), and in the three additional cases only during pacing of the high right atrium at 600 ms, with a delay of 80 ms (range 50–100, IQR 34) (Figure 2). In two patients (#5 and #7), an increase in the delay could be identified during RF application (Figure 3). The median increase after ablation in the interval separating double potentials was 30 milliseconds in sinus rhythm (range 14–76, IQR 33, *p* = 0.003), and 64 ms during atrial pacing (range 22–90, IQR 55, *p* = 0.03). Progressive incremental delay between double potentials during incremental atrial pacing was consistently observed (Figure 3).

A significant correlation was found between TCL at baseline and the delay separating the double potentials post-ablation (R^2^ = 0.64, *p* = 0.006). The median double potential separation in sinus rhythm was 11% of baseline TCL (range 7–15, IQR 5) in inducible patients (baseline), and 19% (range 15–23, IQR 2) in non-inducible patients (post-ablation) (*p* = 0.002). A double potential separation in sinus rhythm of 15% or more of baseline TCL identified non-inducibility with a sensitivity and specificity of 100% (*p* <0.0001).

## 4. Discussion

This study shows for the first time that: (1) mini-electrode bipolar mapping during AVNRT ablation may improve clear identification of so-called slow pathway potentials; (2) visualization of a significant increase in double potentials interval on the ablation line following RF application, typically ≥15% of baseline TCL, is associated with non-inducibility.

**Figure 4:**
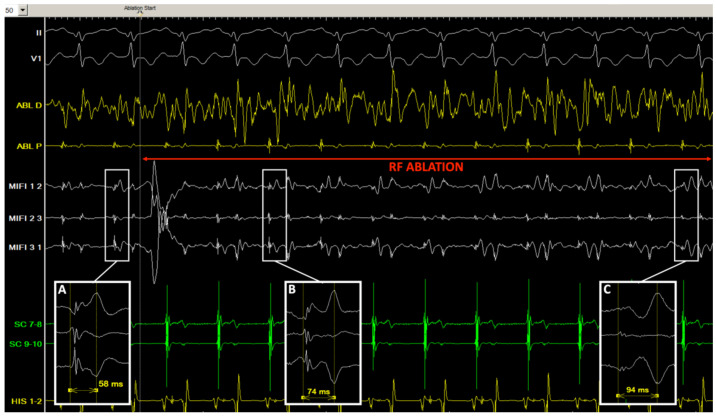
Tracings in patient #7 before and during radiofrequency application. (**A**) Just before ablation, in sinus rhythm, consistent stable double potentials are visualized (separation delay 58 ms) on the mini-electrodes bipolar channels (MIFI 1 2, 2 3, and 3 1) in the slow pathway area; (**B**,**C**) after 1 s (**B**) and 6 s (**C**) of radiofrequency application (30 W), an incremental double potential interval can be monitored on the insulated mini-electrodes recordings (74 and 94 ms), while the conventional distal ablation channel is completely oversaturated with noise (ABL D).

Historically, 2 different slow pathway potentials have been described. Jackman and colleagues identified a sharp potential either in sinus rhythm or retrogradely in most cases, at the cost of very long procedures [3]. In our study, only rapidly identified slow pathway potentials in sinus rhythm were sought, which might explain that such sharp potentials were found in one-fourth of cases. During ablation, this sharp potential consistently turned into a slow slope potential and split from the initial far-field component (Figure 2 and Figure 4). Haissaguerre and colleagues described discrete low dV/dt potentials, either in sinus rhythm or during atrial pacing, which were rapidly identified in two-thirds of our cases [2]. We consistently observed a significant increase in the delay between the first atrial component and that slow pathway potential following radiofrequency ablation.

Identification of a double, split potential on an ablation line has been associated with conduction block in various arrhythmias, especially atrial flutter, but never with AVNRT [4,5]. Double potentials have been well studied in the AV nodal slow pathway region, prior to ablation [6,7]. They would be “caused by asynchronous activation of two large muscle bundles separated by the mouth of the coronary sinus and thought to be a marker for the region between the coronary sinus orifice and the tricuspid annulus, where the slow pathway is frequently found, rather than specific for the slow pathway itself” [6]. We consistently observed, for the first time, double potentials following RF ablation in our series, thanks to the mini-electrodes positioned directly on the distal (RF-delivering) electrode of the ablation catheter. These electrodes have a very small insulated surface, narrow interelectrode spacing, allowing identification of slow pathway potentials, direct local application of current, and eventually visualization of post-ablation double potentials, without moving the catheter or using any other diagnostic catheter with shorter inter-electrode spacing.

**Figure 5:**
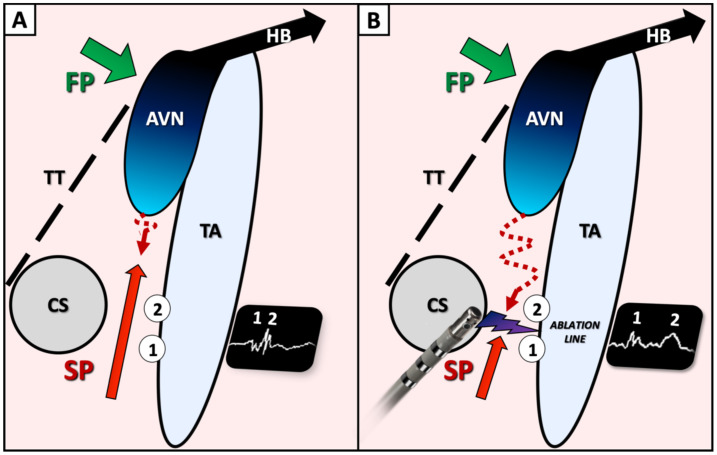
Schematic view of Koch triangle, prior (**A**) and following (**B**) ablation of the slow pathway proposing an explanation of double potentials visualization. During ablation, gradual split of the so-called slow pathway potential (**A**), leading to a double potential can be observed on the ablation line (**B**). The first potential (1) might represent the anterograde activation through the proximal portion of the slow pathway, below the ablation line, while the second potential may represent the late retrograde activation of the distal part of the slow pathway, above the line. FP: fast atrioventricular nodal pathway; SP: slow atrioventricular nodal pathway; CS: coronary sinus ostium; TT: tendon of Todaro; TA: tricuspid annulus; AVN: atrioventricular node; HB: His bundle.

From an electrophysiological standpoint, the first potential might represent the anterograde activation through the proximal portion of the slow pathway, below the ablation line, while the second potential may represent the late retrograde activation of the distal part of the slow pathway, above the line (Figure 5). The interval between potentials may vary according to the site of atrial pacing as previously described [8]. Several clues suggest the critical value of these dual potentials following ablation. The fact that the interval correlates with baseline TCL may be explained by the fact that the AV nodal slow pathway is part of the circuit during tachycardia: the TCL is longer when the conduction over the slow pathway is slower, leading to a longer interval between double potentials following ablation. In patients #5 and #7, a progressive increase in this interval during ablation could actually be observed (Figure 4). Finally, in patient #11, an initial interval of >60 ms (>17% of baseline TCL) post-ablation was obtained and the patient was not inducible. After a short period of observation, the interval decreased to 40 ms (11% of TCL), and the patient was again inducible. Another RF application finally increased permanently the interval up to 64 ms, and the patient was not inducible anymore. This would suggest that achievement of a critical value for the interval between double potentials, ≥15% of baseline TCL in our study, might be used as an ablation endpoint for AVNRT.

It is also totally conceivable that double potentials may not be associated with activation delay in the slow pathway, as previously suggested [9]. However, ablation in the region of the slow pathway would still be increasing separation of these double potentials, and indirectly reflect successful ablation. The delay between double potentials might also depend on the site of ablation: A more proximal ablation site along the slow pathway would be associated with a longer retrograde activation following ablation, and thus a longer delay.

Further trials, with larger samples, are needed to establish a more precise cutoff for the double potential delay and specify the observed mechanisms.

## 5. Conclusions

Mini-electrode bipolar mapping may improve the clear identification of so-called AV nodal slow pathway potentials. It might also allow obtaining, through the identification of double potentials on the ablation line, an electrophysiological endpoint for AVNRT ablation, beyond non-inducibility.

## Figures and Tables

**Table 1:** Patients’ characteristics.

#	Age (Years)	Sex	Symptoms	Cardiac Disease	Treatment	Documentation
1	22	F	Palpitations	0	0	0
2	67	M	Palpitations	0	0	HOLTER
3	43	F	Palpitations	0	0	0
4	61	M	Palpitations	0	0	ECG
5	42	F	Palpitations	0	0	HOLTER
6	71	F	Palpitations	0	Betablocker	ECG
7	60	F	Palpitations	DCM	Betablocker	ECG
8	35	F	Presyncope	0	0	0
9	49	F	Palpitations	0	Verapamil	ECG
10	69	F	Palpitations	0	0	ECG
11	44	F	Palpitations	0	Betablocker	0
12	46	F	Palpitations	0	0	ECG
13	36	F	Presyncope	0	0	0

F, female; M, male; DCM, idiopathic dilated cardiomyopathy.

**Table 2:** Electrophysiological characteristics.

	PRE-ABLATION									POST-ABLATION				
#	AH (ms)	HV (ms)	AV (ms)	VA (ms)	J	H	DP SR	DP AP	TCL (ms)	AH (ms)	HV (ms)	AV (ms)	DP SR	DP AP
1	146	34	353	-	+	-	30	-	300	95	38	-	60	-
2	112	48	400	429	-	-	-	-	270	100	42	340	52	64
3	108	40	286	324	+	-	52	-	350	92	40	333	66	-
4	95	72	375	261	-	+	44	-	310	-	-	-	72	90
5	115	55	261	286	-	+	35	52	380	100	52	273	58	74
6	106	50	300	316	+	+	28	-	410	-	-	-	76	-
7	110	62	333	375	-	+	-	39	415	-	-	333	-	100
8	88	50	600	600	-	-	-	-	300	95	46	333	-	80
9	94	50	316	353	-	+	-	61	230*	85	40	240	30	-
10	72	52	353	-	-	+	45	-	350	90	54	353	63	-
11	118	32	274	316	-	+	-	30	350	110	36	414	62	98
12	86	40	316	353	-	+	-	25	375	90	38	316	76	-
13	164	46	343	350	-	-	-	25	280	-	-	353	-	50

AH, atrio-hisian interval; HV, hisio-ventricular interval; AV, atrio-ventricular refractory period; VA, ventriculo-atrial refractory period; J, Jackman potential identification; H, Haissaguerre potential identification; DP SR, double potential identification in sinus rhythm; DP AP, double potential identification during atrial pacing; TCL, tachycardia cycle length.

## Data Availability

Data can be obtained from the corresponding author upon reasonable request.
